# Dysautonomia and REM sleep behavior disorder contributions to progression of Parkinson’s disease phenotypes

**DOI:** 10.1038/s41531-022-00373-0

**Published:** 2022-08-30

**Authors:** Giulietta Maria Riboldi, Marco J. Russo, Ling Pan, Kristen Watkins, Un Jung Kang

**Affiliations:** 1 Department of Neurology, the Marlene and Paolo Fresco Institute for Parkinson’s Disease and Movement Disorders, New York University Langone Health, New York, NY 10017 USA; 2 NYU Langone Neurosurgery Associates, New York, NY 10016 USA; 3 Icahn School of Medicine at Mount Sinai, New York, NY USA; 4 Department of Neuroscience and Physiology, Neuroscience Institute, The Parekh Center for Interdisciplinary Neurology, New York University Grossman School of Medicine, New York, NY 10016 USA

**Keywords:** Parkinson's disease, Movement disorders

## Abstract

Non-motor symptoms of Parkinson’s disease (PD) such as dysautonomia and REM sleep behavior disorder (RBD) are recognized to be important prodromal symptoms that may also indicate clinical subtypes of PD with different pathogenesis. Unbiased clustering analyses showed that subjects with dysautonomia and RBD symptoms, as well as early cognitive dysfunction, have faster progression of the disease. Through analysis of the Parkinson’s Progression Markers Initiative (PPMI) de novo PD cohort, we tested the hypothesis that symptoms of dysautonomia and RBD, which are readily assessed by standard questionnaires in an ambulatory care setting, may help to independently prognosticate disease progression. Although these two symptoms associate closely, dysautonomia symptoms predict severe progression of motor and non-motor symptoms better than RBD symptoms across the 3-year follow-up period. Autonomic system involvement has not received as much attention and may be important to consider for stratification of subjects for clinical trials and for counseling patients.

## Introduction

Non-motor symptoms often precede the classic motor symptoms of Parkinson’s disease (PD). In particular, rapid eye movement (REM) sleep behavior disorder (RBD) and pure autonomic failure (PAF) have been noted to be prodromal syndromes with high rates of phenoconversion to manifest central neurodegenerative synucleinopathies such as PD, dementia with Lewy bodies (DLB), or multiple system atrophy (MSA)^1–4^. RBD is a parasomnia characterized by recurrent episodes of dream-enactment behavior, including vocalizations and/or complex motor movements, resulting from loss of atonia during REM sleep^5^. Idiopathic RBD (iRBD) is thought to represent an early brainstem manifestation of α-synuclein pathology^6,7^. Clinically, >80% of patients with iRBD will progress to neurodegenerative synucleinopathies within 10–14 years^8,9^. In a large, autopsy-validated cohort of RBD patients, synucleinopathy was determined to be the underlying neuropathology in 94% of cases^10^. Thus, RBD provides an important window to identify patients who are at risk of developing neurodegeneration from synucleinopathy.

PAF, characterized by progressive degeneration of the autonomic nervous system, has also been shown to have high likelihood of phenoconversion to neurodegenerative synucleinopathy^4,11^. Close association of iRBD and autonomic dysfunction has been noted. Studies of patients with iRBD have higher rates of autonomic dysfunction compared to healthy controls^12^ whereas probable RBD is present in the majority of patients with PAF^13^. However, there are conflicting data on the role of autonomic dysfunction in predicting phenoconversion from iRBD to neurodegenerative synucleinopathies, with some prospective studies showing no increased rates of autonomic dysfunction, but other studies showing more severe constipation, erectile and urinary dysfunction in early converters^1,12,14–16^.

On the other hand, a significant proportion of manifest PD patients have no RBD or significant autonomic dysfunction. Therefore, the presence or absence of these non-motor symptoms may indicate different trajectories and possibly disparate pathogenesis of PD. Many studies that have sought to identify clinical subtypes of PD are based on unbiased analyses. Some reported that symptoms including RBD, autonomic dysfunction, cognitive dysfunction, hallucinations and apathy are associated with worse motor function and more severe disease in patients with PD^17,18^. Others have proposed, based on imaging studies, that PD starting with peripheral and lower brainstem symptoms, such as autonomic dysfunction and RBD, may manifest a different pathogenesis from those starting with early central pathology^19–21^. While unbiased cluster analyses provide powerful methods to optimize classification into subtypes for predicting progression for the cohort studied, the multivariate combination of classifiers makes it difficult to replicate across studies and too complex to apply in clinical practice settings^22^.

Therefore, we conducted a hypothesis-driven analysis of the role of RBD and autonomic dysfunction as distinctive traits for PD subtypes and analyzed the interaction of these two classifiers to understand the contribution of each trait to PD severity and progression. We focused on data that can be easily assessed during the office visit to define PD subtypes simply and consistently so that their effects on disease progression can be validated across different cohorts. Such information can provide an informative tool for clinical trial stratification and counseling patients about disease implications and progression.

## Results

### Unsupervised clustering analysis of clinical traits highlights correlation between pRBD and dysautonomia

**Fig. 1:**
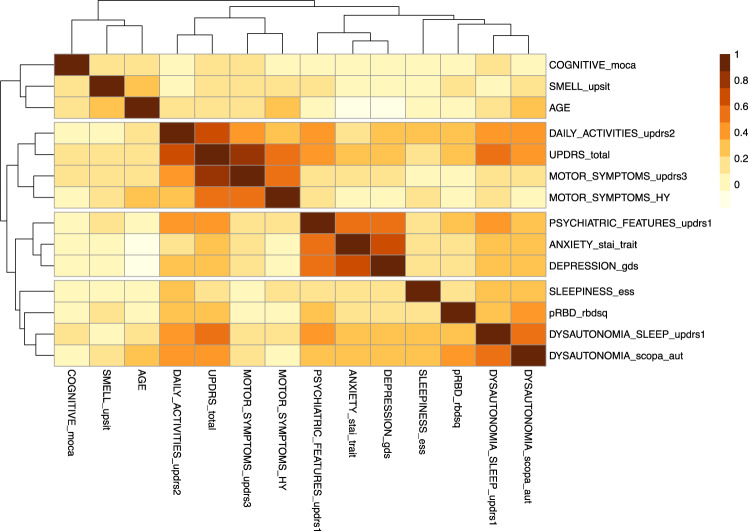
Unsupervised clustering analysis of baseline motor and non-motor traits in PD subjects. Hierarchical clustering of Pearson correlation coefficients of motor and non-motor symptoms in the PPMI cohort (*n* = 423). Color scale (red-orange-yellow) in the heatmap represents degree of correlation between traits (color scale on the right side of the figure). Hierarchical clustering of Pearson correlation of the symptoms based on Euclidian distance is reported on both *x* and *y* axes. Symptoms are clustered in four groups based on unsupervised Pearson correlation. COGNITIVE_moca: Montreal cognitive assessment; SMELL_upsit: University of Pennsylvania smell identification test; PSYCHIATRIC_FEATURES_updrs1: MDS-UPDRS part 1 (question 1–6); DYSAUTONOMIA_SLEEP_updrs1: MDS-UPDRS part 1 (question 7–13); DAILY_ACTIVITIES_updrs2: MDS-UPDRS part 2; UPDRS_total: MDS-UPDRS total score; MOTOR_SYMPTOMS_updrs3: MDS-UPDRS part 3; MOTOR_SYMPTOMS_HY: Hoehn and Yahr scale; ANXIETY_stai_trait: State-Trait Anxiety Inventory (STA-TRAIT); DEPRESSION_gds: Geriatric depression scale; SLEEPINESS_ess: Epworth Sleepiness Scale; pRBD_rbdsq: possible RBD, REM Sleep Behavior Disorder Screening Questionnaire; DYSAUTONOMIA_scopa_aut: Scales for Outcomes in Parkinson’s Disease.

We utilized data from Parkinson’s Progression Marker’s Initiative (PPMI) study cohort, including 423 subjects with PD diagnosis that was unchanged across 3 years of follow-up. Hierarchical clustering based on relative correlation between different baseline symptom domains in subjects with PD revealed four main clusters: cluster 1, comprising motor symptoms (MDS-UPDRS part 3, H&Y stage), activities of daily living (MDS-UPDRS part 2) and MDS-UPDRS total score; cluster 2, comprising non-motor symptoms and demographic features (cognition, olfactory function, and age) with poor correlation with each other; cluster 3, comprising psychiatric features (as assessed by the scores of the GDS, STAI-TRAIT, and MDS-UPDRS part 1 questions 1–6 scales); cluster 4, which includes sleep- and autonomic-related symptoms as assessed by the RBDSQ, SCOPA-AUT, and MDS-UPDRS part 1 questions 7–13 (Fig. 1). There was close association between pRBD and dysautonomia scores (correlation coefficient: pRBD_rbdsq-DYSAUTONOMIA_scopa *R* = 0.92, *p* = 3.25E-06) (Supplementary Table 1a, b).

### Validation of autonomic rating scales with physiologic cardiovascular measures

Prior to further analysis, we sought to validate autonomic symptom questionnaires with available physiologic autonomic data. We assessed correlation between orthostatic blood pressure and heart rate measurements and the cardiovascular sub-scores of SCOPA-AUT (*n* = 421 subjects with available baseline orthostatic data). Neurogenic orthostatic hypotension (nOH, defined as ΔHR/ΔSBP = < 0.5, see Methods) was present in 8% of subjects at baseline, which increased to 13% by year 3. Two-way ANOVA revealed significant difference in SCOPA-AUT scores between nOH and non-nOH (*F*_(1, 1530)_ = 6.415, *p* = 0.0114), and significant differences from baseline to year 3 (*F*_(3, 1530)_ = 4.18, *p* = 0.0058), but pairwise comparisons at baseline or year 3 were modest and non-significant (Tukey). SCOPA-AUT scores are higher in subjects with nOH than those without nOH when baseline and year 3 were pooled (12.5 vs. 10.5, *p* = 0.0106, *t* test). This is consistent with an objective physiologic basis to an otherwise subjective assessment of dysautonomia. Since the initial fraction of subjects with nOH is small and individual sub-traits of the SCOPA-AUT are all mutually positively correlated, the total score was utilized for downstream analysis (Supplementary Fig. 1). Scores from questions 7–13 of the MDS-UPDRS parts 1 also correlated with SCOPA-AUT, reflecting the overlap of non-motor symptom items in these scales (Fig. 1). Therefore, we proceeded with these questionnaire data to specifically test the hypothesis that these clinical features are associated with distinct trajectories of PD.

### Dysautonomia-related symptoms and pRBD are variable across time

We identified 325 subjects within the PPMI cohort with SCOPA-AUT score (as a proxy for dysautonomia state) across the 3 years of study (Supplementary Fig. 3). Based on cutoff scores as previously described (see Methods)^17^, we observed that at baseline the majority (64%) of PD subjects reported symptoms of autonomic dysfunction (Table 2). By year 3, 76% of subjects reported dysautonomia (Table 2). There were 337 subjects with RBDSQ data available across the 3-year follow-up period (Supplementary Fig. 3). We explored whether presence of RBD-related symptoms influences overall phenotype and progression of PD by categorizing the cohort based on RBDSQ question 6 scores from baseline to year 3. This analysis showed that 44% of subjects at baseline have positive pRBD score, which increases to 53% at year 3 (Table 1). The majority of subjects (56%) remained consistently pRBD+ or pRBD− across all time points (24% and 32%, respectively) (Table 1, Supplementary Table 2).

**Table 1:** pRBD symptoms from BL to year 3.

	pRBD	
	+	−
BL	149 (44%)	188 (56%)
Y3	180 (53%)	157 (47%)
Consistent RBSSQ-q6 BL-Y3	82 (24%)	107 (32%)
RBDSQ-q6 ≥1 in at least 1 visit	230 (68%)	/

Proportion of PD cases with positive or negative pRBD symptoms (defined by score ≥1 at RBDSQ-q6 or RBDSQ-q6 = 0) at BL (first row) and at follow-up year 3 (second row). “Consistent RBSSQ-q6 BL-Y3” refers to subjects who had RBDSQ-q6 ≥ 1 (pRBD+) or RBDSQ-q6 = 0 (pRBD−) at all four time points between BL and Y3. The last row refers to subjects who had at least one visit with RBDSQ-q6 ≥ 1 between BL and Y3 (pRBD+). Only subjects with available RBDSQ score at all visits (BL-Y3) were considered in the analysis.

Responses to the RBDSQ across visits were not always consistent within the same subjects. Indeed, 50 (22%) of the subjects who reported positive RBD symptoms at earlier visits reported absence of symptoms at the last visit (year 3) (Supplementary Table 2). It was less common for dysautonomia symptoms to disappear by year 3 (34 subjects [22%] Supplementary Table 3). The comparison between SCOPA-AUT total and sub-scores for subjects who were taking medications with potential autonomic effects vs. those who are not on these medications showed that baseline SCOPA-AUT scores were not different (Supplementary Fig. 4). We also compared changes in SCOPA-AUT scores from visits at baseline to year 1 for those who started symptomatic treatment with PD medications vs. those who remained off of PD medications at year 1. SCOPA-AUT scores showed significant effect of time from baseline to year visits and of medication status (Supplementary Fig. 5). However, there is no interaction between medication status and time, indicating that SCOPA-AUT score increase over 1 year was not differentially affected by medication status (Supplementary Fig. 5). This suggests that other factors, present at baseline, are contributing more to the score increase than the interval start of PD medications (Supplementary Fig. 5). Therefore, overall, we did not find evidence of the significant effect of PD or cardiovascular medication on SCOPA-AUT symptoms at baseline and within a year of starting the PD medications.

### Dysautonomia, but not isolated pRBD, correlates with progression of PD-related motor and non-motor symptoms

We then assessed the predictive value of RBD and dysautonomia symptoms on motor and non-motor symptoms progression. A mixed-effects model for regression of each trait across repeated visits found a large and significant effect of dysautonomia (total SCOPA-AUT score) on progression of motor symptoms (MDS-UPDRS part 3, H&Y score), activities of daily living (MDS-UPDRS part 2), non-motor symptoms (MDS-UPDRS parts 1, anxiety [STAI-TRAIT score], and depression [GDS score]), as well as on the MDS-UPDRS total score. There was no effect of these variables on cognitive impairment (MoCA score), possibly because of the early-stage disease of PPMI cohort, who have minimal cognitive deficit (Table 3). The effect of pRBD alone did not show any significant effect and the interaction of pRBD and dysautonomia only significantly correlated with the MDS-UPDRS part 2 score (Table 3). We did not see significant correlation of baseline SCOPA-AUT scores with DATSCAN progression (Supplementary Fig. 6), consistent with previous data showing poor correlation of change in DATSCAN with changes in motor signs measured by MDS-UPDRS part 3^23^.

Among the sub-scores of the SCOPA-AUT scale, gastrointestinal (GI), sexual (SEX), and pupillomotor (PM) symptoms most correlated with the progression of some of the motor and non-motor symptoms of PD (Supplementary Table 5).

### Characterization of dysautonomia/pRBD subtypes

**Fig. 2:**
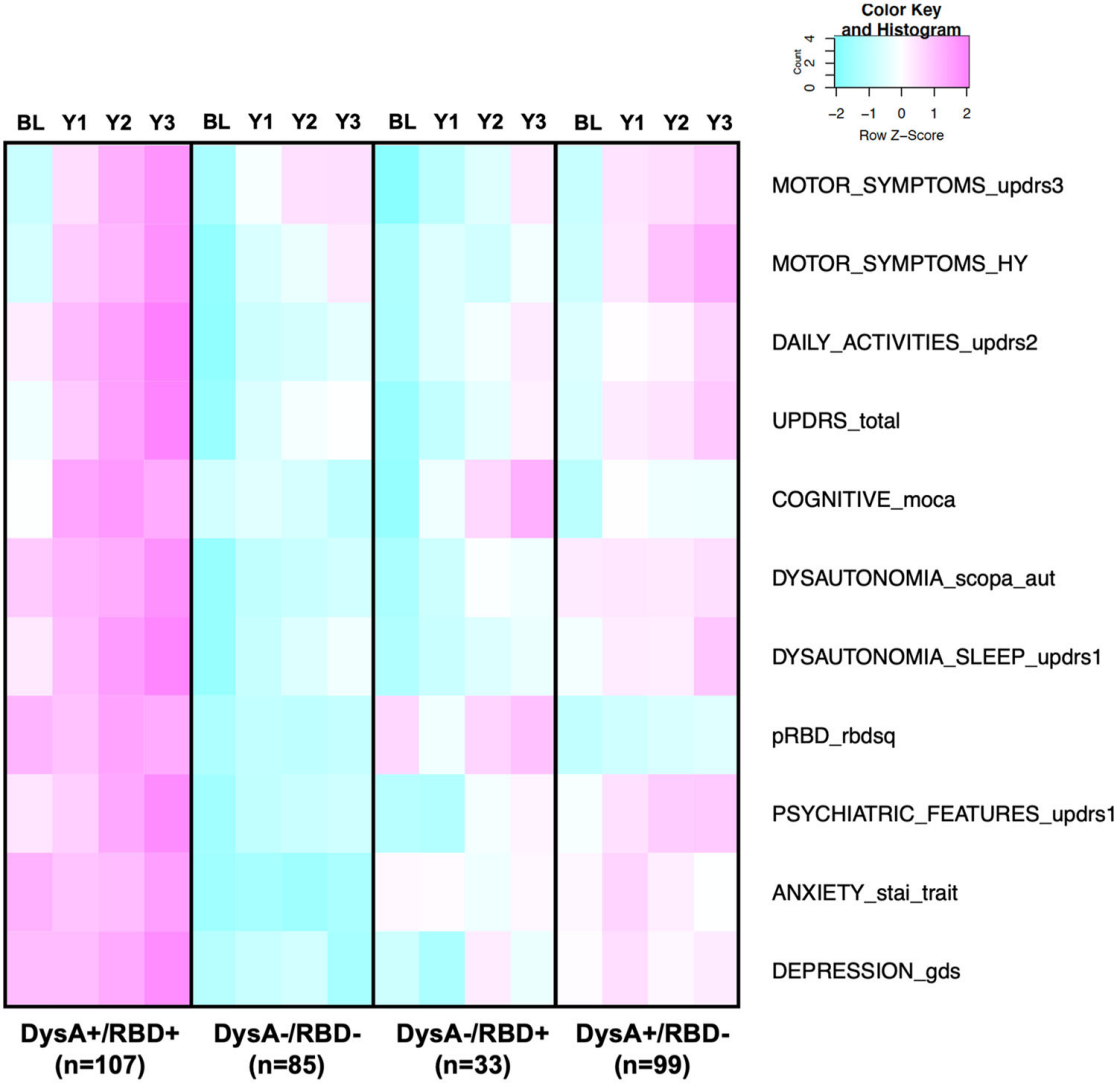
Progression and severity of phenotypical traits across visits in sub-grouped of patients classified based on pRBD and dysautonomia score at baseline. The figure represents the *z* score across visits and groups of the different traits at each time points. pRBD+: subject with RBDSQ question 6 ≥ 1 at BL; pRBD−: subjects with RBDSQ question 6 = 0 at BL; DysA+: subject with SCOPA-AUT score ≥7 at BL; DysA−: subjects with SCOPA-AUT <7 at BL. MoCA scale is expressed as subtracted score (30 - score) for consistency with directionality of other scales.

To further assess the role of RBD− and dysautonomia symptoms in identifying PD subtypes, we subdivided the PD cohort on the basis of dysautonomia status (DysA+ or DysA−) and pRBD status (pRBD+ or pRBD−) at baseline into four groups: DysA+/pRBD+, DysA+/pRBD−, DysA−/pRBD+, and DysA−/pRBD−. For this analysis, we excluded subjects with missing RBDSQ or SCOPA-AUT scores across the 3-year follow-up visits and retained a cohort of 324 subjects (Supplementary Fig. 3). The subgroup with both dysautonomia and pRBD symptoms (DysA+/pRBD+) presented with a more severe pattern of motor and non-motor symptoms (Fig. 2).

Pairwise multiple comparisons between groups confirmed significant difference in non-motor symptoms, particularly between the DysA+/pRBD+ vs. DysA−/pRBD− groups (Supplementary Table 4a). At baseline, dysautonomia (by SCOPA-AUT or MDS-UPDRS part 1 questions 7–13), differences in the anxiety and depression (STAI-TRAIT, GDS, or MDS-UPDRS part 1 questions 1–6 scores), RBD score, activity of daily living (MDS-UPDRS part 2), and MDS-UPDRS total scores were statistically significant between DysA+/pRBD+ vs. DysA−/pRBD−. Difference in motor symptoms scores (H&Y scales) were instead observed only at follow-up year 3 but not at baseline between DysA+/pRBD+ vs. DysA−/pRBD− and Dys−/pRBD+ vs Dys+/pRBD+ (H&Y adjusted *p* value = 0.009 and 0.00058, respectively) (Supplementary Table 4b). With comparisons among all groups, a statistically significant difference was more frequently present when groups were discordant for dysautonomia (i.e., DysA+ vs. DysA− groups), than when discordant for pRBD (Supplementary Table 4a, b).

Motor symptoms (MDS-UPDRS part 3 and H&Y) showed significant progression across all 4 groups, as expected for PD, except for H&Y score in the DysA−/pRBD+ group (Table 4). Cognitive symptoms (MoCA score) progressed only in the DysA+/pRBD+ across visits (*p* = 0.0217) (Table 4). Depression and anxiety scores, which were significantly different between DysA+/pRBD+ and DysA−/pRBD− at both baseline and year 3 (Supplementary Table 4a, b) did not show a significant progression over time (Table 4).

Because of the variability of RBD and dysautonomia symptoms across visits (Supplementary Table 2 and Table 2), analyses were repeated by clustering subjects based on symptoms across visits: pRBD− and DysA− were subjects with RBDSQ-q6 and SCOPA-AUT negative (according to the thresholds defined in the Methods) across all visits (baseline to year 3), while pRBD+ or DysA+ were subjects with *at least one* visit who met the criteria for positive symptoms (pRBD or DysA). When comparing the pattern of distribution of motor and non-motor symptoms among subjects classified according to pRBD and Dysautonomia scores at BL (Fig. 2) and across visits (Supplementary Fig. 2) we noted that progression of the traits was consistent between the two analyses. We also classified them more strictly by defining pRBD+ or DysA+ groups to include only subjects with 3 or more visits showing positive scores for pRBD and dysautonomia and again saw a similar pattern (data not shown). This justifies our use of baseline scores for pRBD and dysautonomia for our initial analysis and strengthens the value of considering the presence of these traits for early prediction of disease progression.

**Table 2:** Dysautonomia from BL to year 3.

	Dysautonomia	
	+	−
BL	207 (64%)	118 (36%)
Y3	248 (76%)	77 (24%)
Consistent SCOPA-AUT BL-Y3	175 (54%)	43 (13%)
SCOPA-AUT ≥7 in at least 1 visit	282 (87%)	/

Proportion of PD cases with positive or negative pRBD symptoms (defined by score ≥7 at SCOPA-AUT or SCOPA-AUT <7) at BL (first row) and at follow-up year 3 (second row). “Consistent SCOPA-AUT BL-Y3” refers to subjects who had SCOPA-AUT ≥7 (DYSAUTONOMIA+) or SCOPA-AUT <7 (DYSAUTONOMIA-) at all four time points between BL and Y3. The last row refers to subjects who had at least one visit with SCOPA-AUT ≥7 between BL and Y3 (DYSAUTONOMIA+). Only subjects with available SCOPA-AUT score at all visits (BL-Y3) were considered in the analysis.

## Discussion

Studies utilizing unbiased subgrouping analyses with data driven approaches have noted that the presence of RBD and dysautonomia reflects a distinct PD subtype often with worse prognosis^17,18,24–31^. However, these unbiased studies include many other factors, such as cognitive deficits. We employed unsupervised hierarchical clustering analysis to confirm the prevailing literature indicating the importance of non-motor symptoms such as RBD and dysautonomia as well as psychiatric symptoms in identifying subgroups with potentially distinct pathophysiology. Since RBD and dysautonomia are also prodromal symptoms with high probability of phenoconversion, we focused on understanding the relative contributions of these two symptom complexes to progression of PD in a well characterized de novo cohort. We leveraged self-assed rating scales, such as the RBDSQ and SCOPA-AUT scales, that can be easily assessed during an office visit and therefore could make identification of PD subtypes more consistent and replicable to inform patient counseling of potential progression.

By employing a mixed-effects model, we came to the unexpected conclusion that dysautonomia is the main driver of clinical progression. RBD is tightly associated with dysautonomia, and therefore may appear to influence progression, as often noted in the literature. This also highlights the heterogeneity of PD subtypes, with one subtype (dysautonomia+ and pRBD+) with more severe motor and non-motor symptoms, possibly due to increased alpha-synuclein load, while recognizing a smaller subgroup without dysautonomia or pRBD that has slower disease progression (Fig. 2). While RBD has been shown to be the most robust risk factor for phenoconversion to central synucleinopathy, our analyses emphasize the predominant effect of dysautonomia as a predictor of symptom progression^1^. We found no statistically significant correlation between pRBD or dysautonomia with cognitive changes (MoCA score) in our analysis, although the progression of cognitive symptoms seemed to have a worse trend in subjects with pRBD more than dysautonomia (Fig. 2, Supplementary Fig. 2, Table 4), suggesting a possible neuropathological correlation. It is important to consider that our cohort included subjects with early-stage PD (within 2 years from diagnosis) and 3-year follow-up. Thus longer longitudinal studies will help further understand the possible stronger correlation between pRBD and cognitive features.

In our study we also found that pRBD symptoms changed throughout the course of the 3-year follow-up period. Reliability and reproducibility of subjective reporting of RBD symptoms has never before been systematically validated. Interestingly, some subjects reported the emergence of RBD symptoms after the baseline visit, while others reported no symptoms during later visits despite initially reporting RBD symptoms. Negative RBD scores in subjects who were previously positive may be interpreted as reduced perception of the symptoms by these subjects as the disease progresses, changes in sleep architecture with time, or the effects of medications started to treat RBD or indirect effects of other medications^32–35^. Future studies correlating clinical, physiological, pathological and imaging data can help elucidate this point. For dysautonomic features, such as gastrointestinal, urinary symptoms, orthostatic hypotension, and sexual dysfunction, our analysis showed worsening SCOPA-AUT scores across follow-up visits in all patients, as expected in PD, although with a certain degree of variability (Table 2 and Supplementary Table 3). In our analysis, we considered the total dysautonomia score, as we showed a positive correlation between all the sub-scores of the scale and consistency with objective physiological data for nOH. This is somewhat contrary to a previous paper noting differential effects of various autonomic symptoms on the overall risk of photoconversion from RBD to neurodegenerative synucleinopathy^1^. The percentage of subject with OH in our cohort was similar to a previous report of an independent early PD cohort^13^. Dysautonomia is an important component in the spectrum of PD manifestations and also suggests peripheral involvement of the disease^19,20^.

As expected for the degenerative nature of PD, a positive rate of progression of motor and non-motor symptoms was observed in all of the subgroups based on RBD or dysautonomia. However, motor, psychiatric, as well as dysautonomia rating scales were consistently higher in subjects in the dysautonomia+/pRBD+ group across visits (Table 3). The PPMI cohort included newly diagnosed and medication-free patients within 2 years of onset of the disease. Thus, disease duration, age or effect of PD medications should not affect theses analyses. Subjects with positive RBD and dysautonomia scores are affected with more severe phenotypes from the initial stages of the disease. Therefore, we can postulate that they represent a distinctive neuropathological phenotype, possibly characterized by more widespread pathology and higher burden of α-synuclein from the very initial phases of the disease.

**Table 3:** Contribution of dysautonomia and pRBD symptoms on the progression of motor and non-motor PD-related symptoms.

	pRBD (*p*)	DYSAUTONOMIA (SCOPA-AUT) (*p*)	pRBD*DYSAUTONOMIA (*p*)
MDS-UPDRS Part 1 (dys_sleep)	0.327	<0.001***	0.773
MDS-UPDRS Part 1 (psychiatric)	0.920	<0.001***	0.303
MDS-UPDRS Part 2 (daily activities)	0.660	<0.001***	0.029*
MDS-UPDRS Part 3	0.197	0.012*	0.308
MDS-UPDRS Total	0.543	<0.001***	0.160
H&Y	0.452	<0.001***	0.142
STAI (anxiety)	0.100	0.003**	0.861
GDS (depression)	0.863	0.002**	0.193
MoCA (cognitive)	0.580	0.643	0.432

Mixed-model for multiple regression was used to assess the contribution of dysautonomia (SCOPA-AUT score), pRBD, and of the interaction between those two traits (pRBD* DYSAUTONOMIA) on motor (MDS-UPDRS part 3, H&Y, MDS-UPDRS part 2) and non-motor symptoms. Non-motor symptoms included cognitive function assessed by MoCA, psychiatric symptoms such as depression by GDS scale and anxiety by STAI-TRAIT scale, and by MDS-UPDRS part 1 questions 1–6 (psy), dysautonomia, as asssessed by MDS-UPDRS part 1 questions 7–13 (dys_sleep), and MDS-UPDRS total score, across visits (BL-Y3). pRBD: binary score (subjects with RBDSQ-q6 = 0 at all visits vs subjects with at least one visit with score >1). ***<0.001; **<0.01; *<0.05. Statistically significant results are in bold face.

Our work has some limitations that may be overcome by future validation studies. First, polysomnography (PSG), which is the gold standard for diagnosis of RBD, was not utilized in the current PPMI dataset^36^. However, using only rating scales facilitates identification of predictors based on instruments that are easily accessible and which may be clinically applied to a wider population. This is also true for defining dysautonomia based on SCOPA-AUT scores. Also, the limited follow-up (up to 3 years) in newly diagnosed patients with PD, may have limited assessment of correlation of dysautonomia and RBD with progression of cognitive symptoms, which usually deteriorate at later stages of PD.

In conclusion, our study shows that dysautonomia is associated with a more severe PD phenotype, possibly corresponding to a distinct neuropathological subtype with more widespread involvement across peripheral and central nervous system locations. These observations have important prognostic value for the counseling of patients presenting to the clinic and for stratification of subjects for observational and therapeutic studies.

## Materials and methods

### Study cohort and data processing

Data were downloaded from the LONI Parkinson Progressive Markers Initiative (PPMI) database on 20th April 2020. PPMI is an international, multi-center, longitudinal observational study that collects comprehensive motor and non-motor data, with the goal of identifying clinically significant biomarkers in de novo PD patients (diagnosed within 2 years) [16]. Each Parkinson’s Progression Markers Initiative (PPMI) site received approval from an ethical standards committee on human experimentation before study initiation and obtained written informed consent from each study participant. Complete descriptions of data collected by the PPMI study can be found at www.ppmi-info.org. We selected only subjects with diagnosis of PD or healthy control subjects enrolled in the study at the moment data were downloaded. Subjects enrolled in the genetic registry or genetic, prodromal, or SWEDD cohorts were not included. We considered only subjects whose diagnosis did not change during a 3-year follow-up period (*n* = 423). Scores from the following rating scales were considered: Movement Disorder Society-Unified PD Rating scale (MDS-UPDRS) total score, MDS-UPDRS part 3, MDS-UPDRS part 2, MDS-UPDRS part 1 (question 1–6, relevant to cognitive and psychiatric features), MDS-UPDRS part 1 (question 7 to 13, relevant to sleep-related and autonomic symptoms), Hoehn and Yahr scale (H&Y), University of Pennsylvania smell identification test (UPSIT), Montreal cognitive assessment (MoCA), Scales for Outcomes in PD-Autonomic dysfunction (SCOPA-AUT), REM Sleep Behavior Disorder Screening Questionnaire (RBDSQ), Epworth Sleepiness Scale (ESS), Geriatric depression scale (GDS), State-Trait Anxiety Inventory (STAI), heart rate (HR), and blood pressure (SBP). For these scales, higher score corresponds to more severe phenotype, except for the MoCA score, and so we used an inverse score (maximum score - recorded score) in the analysis. We considered scores at the baseline visit (BL), and follow-up visits at year 1, year 2, and year 3. Missing values were imputed with the Multivariate Imputation by Chained Equations (MICE) package for R (v3.8.0) and VIM (v5.1.1) package^37^. Data were analyzed and visualized with R (3.6.0) and RStudio (1.2.1335), Python (3.9.5), and Graphad Prism (9.3.0). Numbers of subjects included in the various analyses are summarized in Supplementary Fig. 3.

### Key definitions for determination of RBD and autonomic phenotypes

Self-assessment questionnaires, such as the RBD screening questionnaire (RBDSQ) and the Scales for Outcomes in PD-Autonomic questionnaire (SCOPA-AUT), were used to determine the phenotypes for RBD and autonomic dysfunction, respectively. These are tools that can be easily performed in an ambulatory setting, and are reproducible. There is high correlation of PSG-proven RBD with the RBD questionnaire results, although with variability^30–33^. Since PSG is often performed only once, the RBD questionnaire may be more sensitive to detect RBD, particularly for question 6, which assesses REM sleep behaviors, though is less specific^34,35^. Most studies have defined these subjects with subjective dream-enactment behavior without PSG confirmation of REM atonia as having probable RBD (pRBD), and we will use the term pRBD to be consistent with the literature. For autonomic symptoms, our analysis is based on the SCOPA-AUT, which includes cardiovascular symptoms (e.g., neurogenic orthostatic hypotension, orthostatic intolerance, syncope), gastrointestinal symptoms (e.g., constipation), thermoregulatory symptoms (hyperhidrosis, heat intolerance), and genitourinary symptoms (e.g., erectile dysfunction, urinary dysfunction with incontinence and/or retention). The total scores higher than 7, which is 75 percentile of the SCOPA-AUT total scores of control group were defined as indicating the presence of dysautonomia as defined by a previous study^17,23,24,26^.

### Clinical traits correlation

Correlation between motor and non-motor traits (considering the following rating scales: MDS-UPDRS total score, MDS-UPDRS part 3, MDS-UPDRS part 2, MDS-UPDRS part 1 (question 1–6) relevant to cognitive and psychiatric features (MDS-UPDRS1_psic), MDS-UPDRS part 1 (question 7 to 13) relevant to sleep-related and autonomic symptoms (UPDRS part 1_dys RBD), HY, UPSIT, MoCA, SCOPA-AUT, RBDSQ, ESS, GDS, STAI) (Fig. 1) and SCOPA-AUT sub-scores (gastrointestinal, urologic, cardiovascular, thermoregulatory, sexual, pupillomotor) (Supplementary Fig. 1) was performed through Pearson’s correlation analysis and hierarchical clustering of symptoms based on Euclidian distance. Missing values were imputed with MICE and VIM (v5.1.1) package, as detailed above^37^.

### Determination of neurogenic orthostatic hypotension status

Presence of neurogenic vs. non-neurogenic orthostatic hypotension was determined from a metric validated in similar population of PD patients, and using corroborating physiologic measures^11,38,39^. Neurogenic orthostatic hypotension (nOH) was determined from the ratio of heart rate change to systolic blood pressure change from supine-to-standing transition (HR_standing_–HR_supine_)/(SBP_standing_–SBP_supine_) = ΔHR/SBP, with ΔHR/ΔSBP = < 0.5 most consistently corresponding to neurogenic forms of OH within the physiogically validated cohort^11^. The ratio was calculated only for subjects with orthostatic hypotension, as defined by ΔSBP ≤ −20 or ΔDBP ≤ −10 mmHg.

### Dysautonomia/pRBD−based group characterization

We classified subjects as probable RBD positive (pRBD+) or probable RBD negative (pRBD−) based on the score of question 6 of the RBDSQ (RBDSQ-q6), as previously reported to have high sensitivity and specificity^40,41^: pRBD+ corresponded to a score greater or equal to 1, pRBD− to a score of 0 across visits from baseline to year 3 (Supplementary Table 2). Subjects were also classified according to cumulative SCOPA-AUT score for dysautonomia. Since a consensus cutoff score is not available for determining dysautonomia based on SCOPA-AUT, we considered greater than 75th percentile of the SCOPA-AUT scores of the pRBD− control population at BL as those with abnormal autonomic function similar to previous publications^17,18,41,42^. DysA+ corresponded to subjects with the total SCOPA-AUT score equal or greater than 7, DysA− were subject with SCOPA-AUT less than 7 across visits from baseline to year 3 (Supplementary Table 3). We considered visits until follow-up year 3 because of increased missingness of the data in the later follow-up visits. Representation of trait progression in the DysA/pRBD−based subgroups for the different traits was obtained with a heatmap of the *z* score of the means of the different traits across timepoints between groups.

Medications were classified by manually parsing the concomitant medications log and assigning each medication to one of the 18 classes indicated in Supplementary Fig. 4. Antihypotensives, including fludrocortisone, midodrine, or droxidopa were not being taken at baseline by any subjects. PD medications are considered separately, though no prescription PD medications were taken at baseline by any subject, as expected. Potential active autonomic medications are of the following classes: α1-antagonists, β-blockers, calcium channel blockers, diuretics, ACE-inhibitors or angiotensin-receptor blockers, vasodilators, tricyclic antidepressants, SSRIs or SNRIs, phosphodiesterase inhibitors, antipsychotics, benzodiazepines, baclofen, narcotics, barbiturates, anticholinesterase inhibitors, anticholinergics, calcium channel α2δ-subunit inhibitors (gabapentin or pregabalin), α2 agonists, and antihypotensives (fludrocortisone, midodrine, droxidopa). Total scores and sub-scores were compared with Mann–Whitney *U* test (*n* = 124 for ‘No Autonomic Med’ group, and *n* = 258 for ‘Taking Potential Autonomic Med’ group).

Comparison between total SCOPA-AUT scores at baseline and 1 year in PD subjects who started PD medication and those who remained off of medication until after 1 year was assessed with two-way ANOVA and Holm-Šídák’s post hoc test or Mann–Whitney test (Supplementary Fig. 5). Linear regression of DaT-SPECT specific binding ratio (ΔSBR) vs. baseline SCOPA-AUT scores of ipsilateral and contralateral caudate and putamen was performed (Supplementary Fig. 6). Subjects were clustered in four groups based on pRBD and dysautonomia scores (RBDSQ-q6 and SCOPA-AUT), as detailed above. *Z* score of the mean scores of rating scales for the different traits was calculated for each group at the different timepoints (from baseline to year 3 follow-up) (Fig. 2, Supplementary Fig. 2). Representation of trait progression in the DysA/pRBD-based subgroups for the different traits was obtained with a heatmap of the *z* score of the means of the different traits across timepoints between groups (Fig. 2, Supplementary Fig. 2).

To compare the four subgroups of subjects, pairwise multiple comparison and Bonferroni post hoc correction was performed for each trait within subgroups at baseline and at year 3 follow-up visit (Supplementary Table 4a and b).

To assess the significance of the progression of scores of the different traits across years of follow-up in the different subgroups ANOVA test was performed, considering a cutoff of *p* < 0.05 for significance (Table 4).

**Table 4:** Progression of trait across visits.

	Dysautonomia (scopa_aut)	pRBD (rbdsq)	Psychiatric features (updrs1)	Motor symptoms (updrs3)	UPDRS total	Anxiety (stai_trait)	Depression (gds)	Cognitive (MoCA)	Motor symptoms (H&Y)	Dysautonomia sleep (updrs1)	Daily activities (updrs2)
DysA+ RBD+	0.000203***	0.145	0.00225**	5.36e-09***	3.44e-12***	0.815	0.483	0.0217*	4.8e-07***	2.18e-06***	1.76e-06***
DysA− RBD−	1.14e-07***	0.192	0.0961	0.000336***	1.11e-07***	0.991	0.602	0.769	0.000434***	1.3e-06***	2.82e-06***
DysA− RBD+	6.04e-06***	0.0298*	0.0444*	0.0134*	0.000131***	0.99	0.369	0.0546	0.54	0.0717	0.000317***
DysA+ RBD−	0.705	0.0368*	0.133	0.000169***	1.21e-06***	0.752	0.805	0.188	9.47e-06***	0.000978***	0.000662***

Variance of the progression of the score of each trait per each clinical subgroup (DysA+ RBD+, DysA− RBD−, DysA− RBD+, DysA+ RBD−) was calculated with ANOVA test. ***<0.001; **<0.01; *<0.05. Clinical subgroups were based on RBDSQ and SCOPA-AUT score at baseline, as detailed in the manuscript. Statistically significant results are in bold face.

### pRBD and dysautonomia correlation with trait progression

In order to assess the specific correlation between pRBD and dysautonomia with the other traits, regression analysis was performed through mixed-effects model accounting for pRBD as binomial value (RBDSQ-q6 = 0 vs RBDSQ-q6 ≥ 1), dysautonomia (SCOPA-AUT cumulative score), and interaction between these two traits was utilized for regression analysis of multiple variables with repeated time points between baseline and follow-up visit 3 (Table 3).

Correlation analysis between SCOPA-AUT sub-scores (GI, CV, URINARY, THERM, PM, SEX) and their interaction with pRBD score were assessed (Supplementary Table 5).

## Supplementary information

supplementary figures

## Data Availability

The data utilized in this work are available at www.ppmi-info.org, previous authorized accessed.

## Code availability

Custom code utilized for the analyses will be available upon reasonable request and as assessed by the authors of this manuscript.
