# Author Correction: Dysautonomia and REM sleep behavior disorder contributions to progression of Parkinson’s disease phenotypes

**DOI:** 10.1038/s41531-023-00463-7

**Published:** 2023-02-04

**Authors:** Giulietta Maria Riboldi, Marco J. Russo, Ling Pan, Kristen Watkins, Un Jung Kang

**Affiliations:** 1 Department of Neurology, the Marlene and Paolo Fresco Institute for Parkinson’s Disease and Movement Disorders, New York University Langone Health, New York, NY 10017 USA; 2 NYU Langone Neurosurgery Associates, New York, NY 10016 USA; 3 Icahn School of Medicine at Mount Sinai, New York, NY USA; 4 Department of Neuroscience and Physiology, Neuroscience Institute, The Parekh Center for Interdisciplinary Neurology, New York University Grossman School of Medicine, New York, NY 10016 USA

**Keywords:** Parkinson's disease, Movement disorders

Correction to: *npj Parkinson’s Disease* 10.1038/s41531-022-00373-0, published online 30 August 2022

In this article in the legend for figure 2 the text “6 ≥ 1 in at least one data point (BL to Y3); pRBD−: subjects with RBDSQ question 6 = 0 at all data points (BL to Y3); DysA+: subject with SCOPA-AUT score ≥7 in at least one data point (BL to Y3); DysA−: subjects with SCOPA-AUT” should have read “6 ≥ 1 at BL; pRBD−: subjects with RBDSQ question 6 = 0 at BL; DysA+: subject with SCOPA-AUT score ≥7 at BL; DysA−: subjects with SCOPA-AUT <7 at BL.” The original article has been corrected.

