# Supplementary material for: Dysautonomia and REM sleep behavior disorder contributions to progression of Parkinson’s disease phenotypes
Source: NPJ Parkinsons Dis. 2022 Aug 30;8:110. doi: 10.1038/s41531-022-00373-0 (PMC9427762; doi:10.1038/s41531-022-00373-0)
Supplement: Supplementary file 1 — supplementary figures [file 41531_2022_373_MOESM1_ESM.pdf]

Supplementary Figures

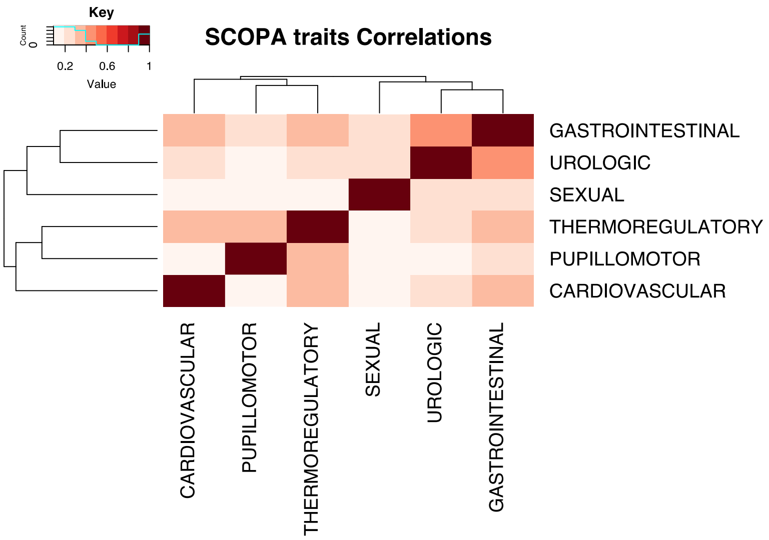

Supplementary Figure 1. Correlation among dysautonomia sub-scores.

Heatmap shows unsupervised clustering of the different sub-scores (urologic, cardiovascular, gastrointestinal, thermoregulation, sexual function, pupillomotor) of the SCOPA-AUT scale. All the sub-scores show positive correlations among one another.

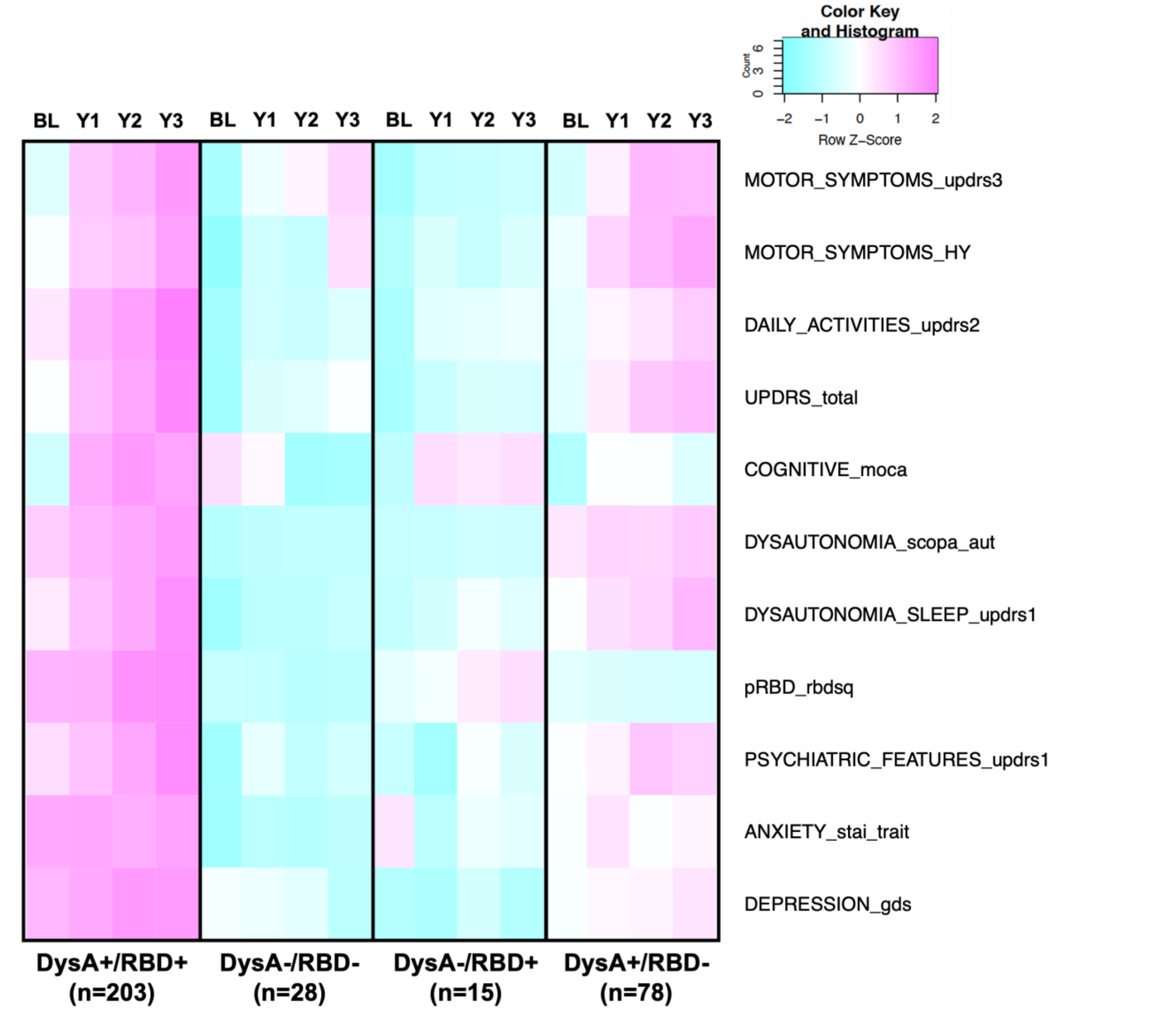

Supplementary Figure 2. Progression and severity of phenotypic traits across visits in sub-grouped of patients classified based on RBD and dysautonomia scores across visits (related to Figure 2). The figure represents the z-score across visits and groups of the different traits at each time points. RBD+: subject with RBDSQ question 6 ≥ 1 for at least one visit (BL to Y3); RBD -: subjects with RBDSQ question 6 = 0 at all visits (BL to Y3); DysA+: subject with SCOPA-AUT score ≥ 7 for at least one visit (BL to Y3); DysA -: subjects with SCOPA-AUT < 7 at all visits (BL to Y3). MOCA scale is expressed as subtracted score (30 - score) for consistency of directionality with those of other scales.

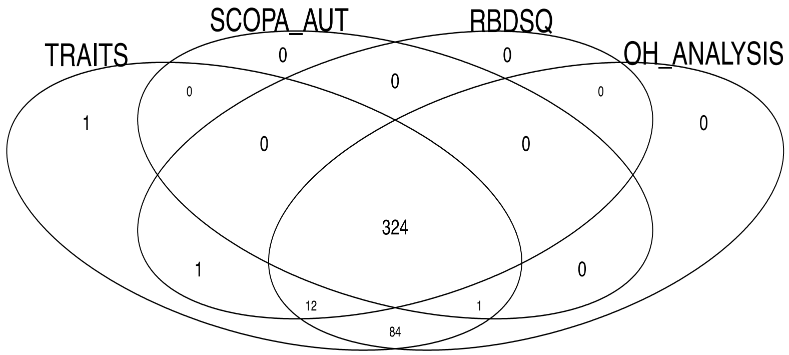

CLASS	NUMBER OF SUBJECTS
TOTAL SUBJECTS (PD, NO CHANGE IN DIAGNOSIS)	423
SUBJECTS FOR OH ANALYSIS	421
SUBJECTS WITH REPORTED RBDSQ BL-Y3	337
SUBJECTS WITH REPORTED SCOPA-AUT BL-Y3	325

Supplementary Figure 3. Sub-cohort of subjects.

The table summarizes the total number and the Venn diagram shows the overlap of subjects included in each analysis as explained in the method section. The differences in the total numbers are due to the missing data.

a

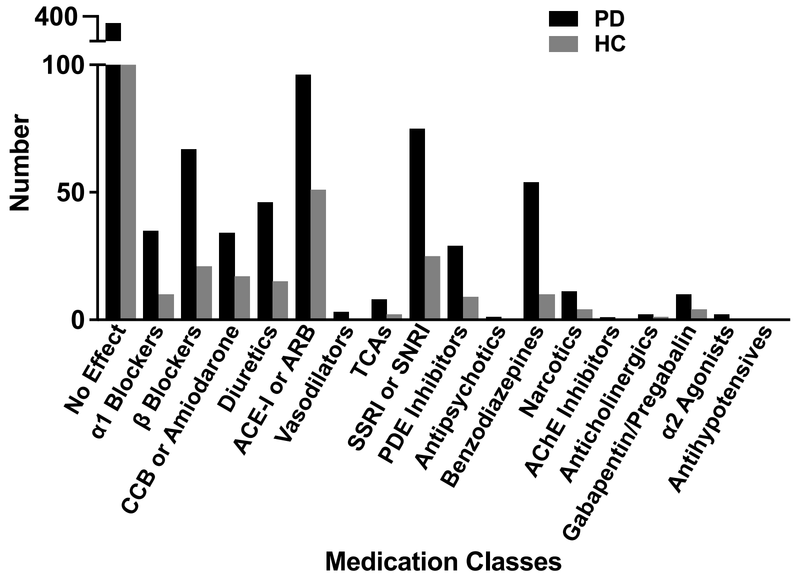

b

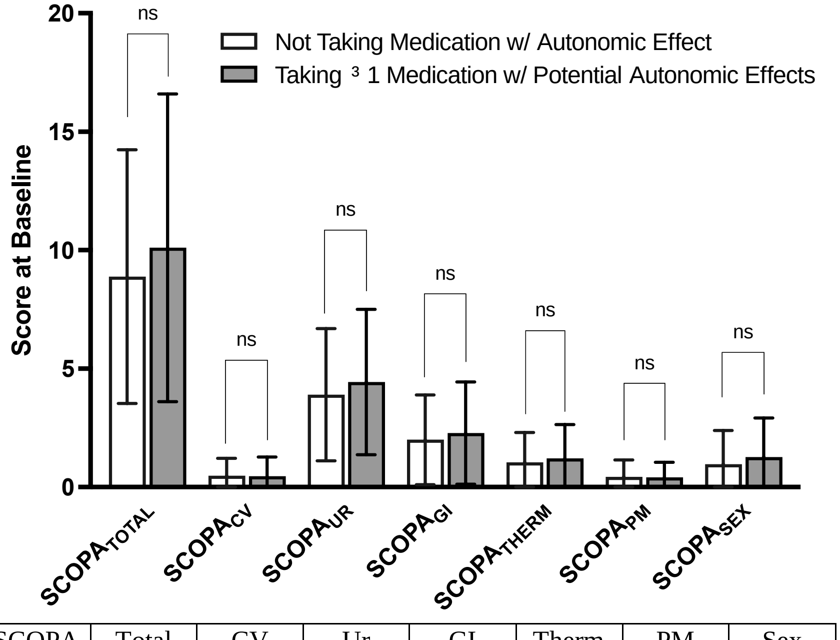

SCOPA	Total	CV	Ur	GI	Therm	PM	Sex
p values	0.128	0.781	0.108	0.317	0.419	0.804	0.100

Supplementary Figure 4. SCOPA score and medication use.

a. Classifications and numbers of medications with potential autonomic effects being taken by PD and HC subjects at baseline in PPMI. Medications were classified by manually parsing the concomitant medications log and assigning each medication to one of the 18 classes indicated. Antihypotensives,

including fludrocortisone, midodrine, or droxidopa were not being taken at baseline by any subjects. PD medications are considered separately, though no prescription PD medications were taken at baseline by any subject, as expected. Medications taken by HC subjects at baseline are shown for reference. **b.** Baseline SCOPA-AUT total scores and sub-scores for PD subjects who were not taking medications from any class with potential autonomic effects. Total scores and sub-scores were compared with Mann-Whitney U test (p values are shown below respective categories, n = 124 for ‘No Autonomic Med’ group, and n = 258 for ‘Taking Potential Autonomic Med’ group).

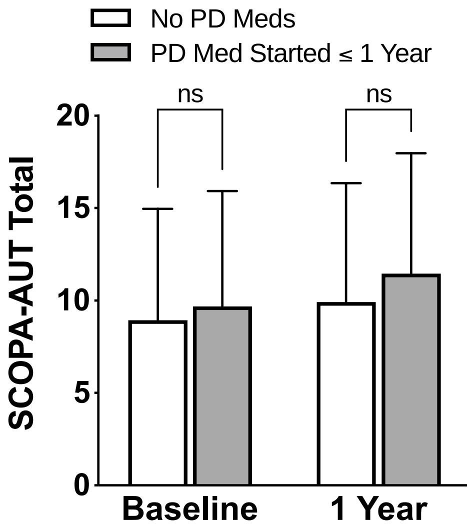

ANOVA	%var	SS	df	MS	$F_{(n, d)}$	p value
**Interaction**	0.082	25.47	1	25.47	$F_{(1, 757)}$ = 0.6392	0.4243
**Time**	1.02	315.5	1	315.5	$F_{(1, 757)}$ = 7.917	0.0050
**PD Meds Status**	0.701	216.6	1	216.6	$F_{(1, 757)}$ = 5.436	0.0200
Residual		30170	757	39.85		

Supplementary Figure 5. SCOPA-AUT progression is not affected by start of PD medications. Total SCOPA-AUT scores at baseline (BL) and 1 year (Y1) were determined for PD subjects who started PD medication (gray boxes) and those who remained off of medication until after 1 year. Two-way ANOVA indicates significant variation between BL and Y1 ($F_{[1, 757]}$ = 7.917, p = 0.005), between medication groups ($F_{[1, 757]}$ = 5.436, p = 0.0200), but without significant interaction ($F_{[1, 757]}$) = 0.6392, p = 0.4243) between time and medication status. *Post hoc* comparisons between those starting medications and those off PD meds were not significant (PD meds vs. no meds, p = 0.2653, p = 0.0620, BL and Y1, respectively; Holm-Šídák's *post hoc* test). ANOVA results shown in table below. Abbreviations: %var, percent total variation; SS, sum of squares; df, degrees freedom; MS, mean squares.

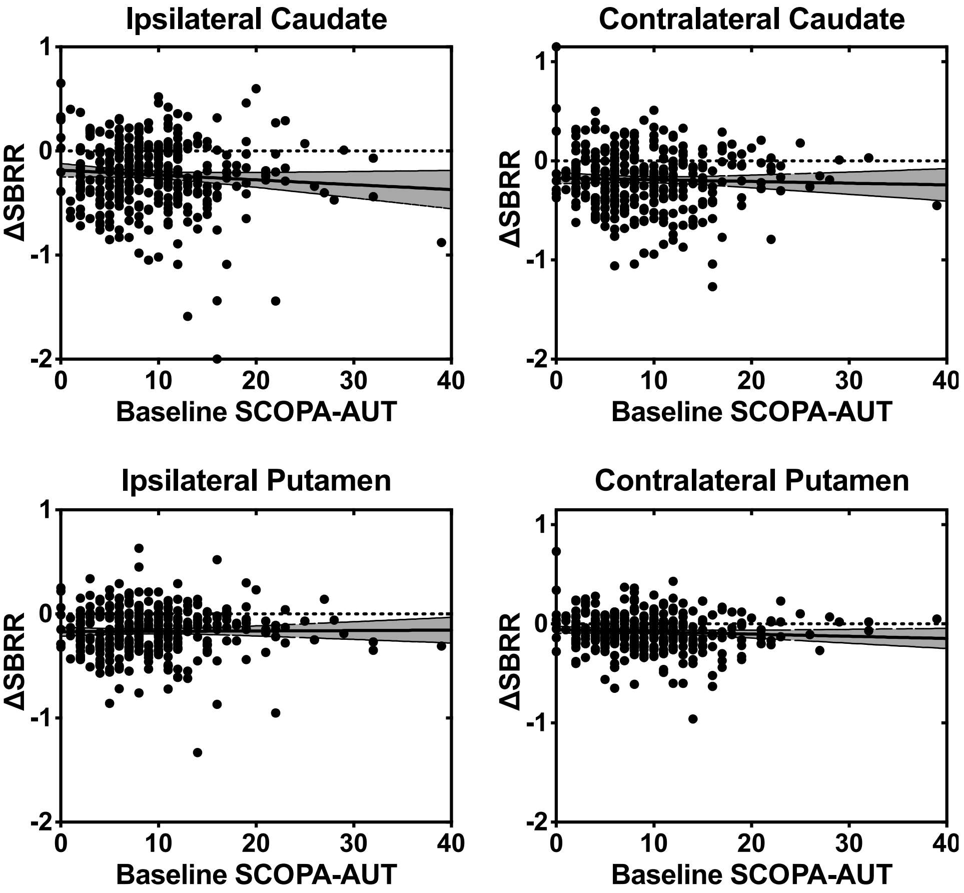

Supplementary Figure 6. Baseline SCOPA-AUT total scores do not predict progression of quantitative DaT-SPECT measures. Baseline SCOPA-AUT totals are plotted against the change (Δ) from baseline to 1 year of DaT-SPECT specific binding ratio (SBR) for each area of the ipsilateral or contralateral striatum. Linear regression of ΔSBR vs. baseline SCOPA-AUT scores *did not* reveal meaningful correlations for ipsilateral caudate (R^2 = 0.00672, p = 0.122), contralateral caudate (R^2 = 0.00116, p = 0.522), ipsilateral putamen ($R^2 = 9.18 \times 10^{-5}$, p = 0857), or contralateral putamen (R^2 = 0.00502, p = 0.182). N = 357 for all comparisons. Gray areas represent 95% confidence interval of linear best fit.

Supplementary Tables

a

	PSYCHIATRIC_FEATURES _updrs1	DYSAUTONOMIA_SLEEP _updrs1	DAILY_ACTIVITIES _updrs2	UPDRS_total	MOTOR_SYMPTOMS _updrs3	MOTOR_SYMPTOMS_ HY	COGNITIVE_ moca	pRBD_rbds q	DYSAUTONOMIA _scopa_aut	SMELL_ upsit	ANXIETY_ stai_trait	DEPRESSION _gds	SLEEPINESS _ess	AGE
PSYCHIATRIC_FEATURES_updrs1	1	0.765160333	0.523127753	0.51045135	-0.132013281	-0.22246696	-0.6055282	0.57645799	0.602422907	-0.6258	0.975771	0.954896067	0.47629577	-0.7885463
DYSAUTONOMIA_SLEEP_updrs1	0.765160333	1	0.853363253	0.66519985	0.107929777	-0.011025365	-0.4646029	0.81057465	0.921720516	-0.5481	0.758545	0.757711089	0.78256071	-0.4211689
DAILY_ACTIVITIES_updrs2	0.523127753	0.853363253	1	0.84708522	0.418042057	0.365638767	-0.2795595	0.6798684	0.784140969	-0.4084	0.439427	0.4224425	0.71223858	-0.2048458
UPDRS_total	0.510451354	0.665199852	0.847085221	1	0.683516484	0.607261094	-0.1434893	0.31868132	0.462046484	-0.2911	0.420242	0.38021978	0.3722476	-0.1672168
MOTOR_SYMPTOMS_updrs3	-0.132013281	0.107929777	0.418042057	0.68351648	1	0.904290976	0.50331616	-0.2087912	-0.019801992	0.2624	-0.222222	-0.26153846	-0.1784145	0.51045135
MOTOR_SYMPTOMS_HY	-0.22246696	-0.011025365	0.365638767	0.60726109	0.904290976	1	0.39116237	-0.3542356	-0.128854626	0.18543	-0.335903	-0.38503874	-0.0837928	0.48678414
COGNITIVE_moca	-0.605528189	-0.464602907	-0.279559547	-0.14348926	0.503316164	0.39116237	1	-0.4569581	-0.468510861	0.69435	-0.653042	-0.67550327	-0.6692494	0.69061153
pRBD_rbdsq	0.576457995	0.810574654	0.679868398	0.31868132	-0.208791209	-0.354235638	-0.4569581	1	0.919692526	-0.3969	0.59186	0.621978022	0.75330579	-0.4048407
DYSAUTONOMIA_scopa_aut	0.602422907	0.921720516	0.784140969	0.46204648	-0.019801992	-0.128854626	-0.4685109	0.91969253	1	-0.4238	0.613436	0.638064192	0.84454296	-0.2907489
SMELL_upsit	-0.625829339	-0.548066633	-0.408389516	-0.29107105	0.262404964	0.185430915	0.69435259	-0.3969151	-0.423842092	1	-0.636867	-0.60860311	-0.5513816	0.75938375
ANXIETY_stai_trait	0.975770925	0.758545114	0.439427313	0.42024228	-0.222222357	-0.335903084	-0.6530423	0.59185954	0.613436123	-0.6369	1	0.990099609	0.49393635	-0.7885463
DEPRESSION_gds	0.954896067	0.757711089	0.4224425	0.38021978	-0.261538462	-0.385038737	-0.6755033	0.62197802	0.638064192	-0.6086	0.9901	1	0.50000121	-0.7612766
SLEEPINESS_ess	0.476295769	0.782560706	0.712238581	0.3722476	-0.17841453	-0.083792774	-0.6692494	0.75330579	0.844542961	-0.5514	0.493936	0.500001213	1	-0.3461965
AGE	-0.788546256	-0.421168944	-0.204845815	-0.16721682	0.510451354	0.486784141	0.69061153	-0.4048407	-0.290748899	0.75938	-0.788546	-0.76127659	-0.3461965	1

b

	PSYCHIATRIC_FEATURES _updrs1	DYSAUTONOMIA_SLEEP _updrs1	DAILY_ACTIVITIES _updrs2	UPDRS_total	MOTOR_SYMPTOMS _updrs3	MOTOR_SYMPTOMS _HY	COGNITIVE_ moca	pRBD_rbdsq	DYSAUTONOMIA _scopa_aut	SMELL_upsit	ANXIETY_ stai_trait	DEPRESSIO N_gds	SLEEPINESS _ess	AGE
PSYCHIATRIC_FEATURES_updrs1	NA	0.001428667	0.054913372	0.062172314	0.652801472	0.444602173	0.02174275	0.03094108	0.022612117	0.016664349	2.77E-09	1.10E-07	0.0851166	0.0008043
DYSAUTONOMIA_SLEEP_updrs1	0.001428667	NA	0.000103853	0.009429579	0.713424472	0.970160038	0.09417902	0.000437507	2.80E-06	0.042451573	0.001662	0.00169303	0.0009377	0.13367993
DAILY_ACTIVITIES_updrs2	0.054913372	0.000103853	NA	0.00013166	0.136894043	0.198574994	0.33305401	0.007470806	0.000900902	0.14714071	0.115922	0.13238549	0.004263	0.48236214
UPDRS_total	0.062172314	0.009429579	0.00013166	NA	0.007036744	0.021268686	0.62457466	0.266784961	0.096246251	0.3126724	0.134627	0.17990615	0.1899672	0.56774011
MOTOR_SYMPTOMS_updrs3	0.652801472	0.713424472	0.136894043	0.007036744	NA	9.00E-06	0.0665482	0.473778837	0.946430404	0.364767235	0.445116	0.36641114	0.5416927	0.06217231
MOTOR_SYMPTOMS_HY	0.444602173	0.970160038	0.198574994	0.021268686	9.00E-06	NA	0.16666609	0.214000153	0.660648999	0.525641122	0.240328	0.17399452	0.7758025	0.0775242
COGNITIVE_moca	0.02174275	0.094179019	0.333054015	0.62457466	0.0665482	0.166666093	NA	0.100454229	0.091078872	0.005862005	0.011335	0.00801716	0.0088533	0.00624881
pRBD_rbdsq	0.03094108	0.000437507	0.007470806	0.266784961	0.473778837	0.214000153	0.10045423	NA	3.25E-06	0.159967756	0.025766	0.01755032	0.0018668	0.15103236
DYSAUTONOMIA_scopa_aut	0.022612117	2.80E-06	0.000900902	0.096246251	0.946430404	0.660648999	0.09107887	3.25E-06	NA	0.130972687	0.019643	0.01407393	0.0001445	0.31323286
SMELL_upsit	0.016664349	0.042451573	0.14714071	0.3126724	0.364767235	0.525641122	0.00586201	0.159967756	0.130972687	NA	0.014313	0.02090695	0.0409669	0.00163054
ANXIETY_stai_trait	2.77E-09	0.001661638	0.11592222	0.134627108	0.445116051	0.240328391	0.01133493	0.025766278	0.01964256	0.014312879	NA	1.33E-11	0.0726295	0.0008043
DEPRESSION_gds	1.10E-07	0.001693033	0.132385486	0.179906146	0.366411137	0.173994519	0.00801716	0.017550315	0.014073931	0.020906945	1.33E-11	NA	0.0686542	0.00156204
SLEEPINESS_ess	0.085116635	0.000937732	0.00426297	0.18996724	0.541692676	0.775802461	0.00885327	0.001866766	0.000144512	0.040966873	0.07263	0.06865423	NA	0.22531289
AGE	0.000804301	0.133679926	0.48236214	0.567740115	0.062172314	0.077524195	0.00624881	0.151032358	0.313232857	0.001630538	0.000804	0.00156204	0.2253129	NA

Supplementary Table 1. Pearson correlation of PD related traits in the PPMI cohort.
a) Correlation coefficient (R value) and **b)** P-values of the Pearson correlation of the unsupervised clustering analysis of baseline motor and non-motor traits in PD subjects (Figure 1). COGNITIVE_moca: Montreal cognitive assessment; SMELL_upsit: University of Pennsylvania smell identification test; PSYCHIATRIC_FEATURES_updrs1: MDS-UPDRS part 1 (question 1-6); DYSAUTONOMIA_SLEEP_updrs1: MDS-UPDRS part 1 (question 7 to 13); DAILY_ACTIVITIES_updrs2: MDS-UPDRS part 2; UPDRS_total: MDS-UPDRS total score; MOTOR_SYMPTOMS_updrs3: MDS-UPDRS part 3; MOTOR_SYMPTOMS_HY: Hoehn and Yahr scale; ANXIETY_stai_trait: State-Trait Anxiety Inventory (STA-TRAIT); DEPRESSION_gds: Geriatric depression scale; SLEEPINESS_ess: Epworth Sleepiness Scale; pRBD_rbdsq: possible RBD, REM Sleep Behavior Disorder Screening Questionnaire; DYSAUTONOMIA_scopa_aut: Scales for Outcomes in Parkinson's Disease.

	pRBD by RBDSQ-q6			
# subjects	BL	YEAR 1	YEAR 2	YEAR 3
82	+	+	+	+
6	+	+	-	+
9	+	-	-	+
23	+	-	+	+
107	-	-	-	-
8	-	+	+	-
6	-	+	-	-
7	-	-	+	-
6	+	+	+	-
8	+	+	-	-
7	+	-	-	-
8	+	-	+	-
13	-	+	+	+
7	-	+	-	+
23	-	-	-	+
17	-	-	+	+

Supplementary Table 2.

PD subjects were grouped based on their score of RBDSQ-q6 across visits (BL to Year 3): (+) indicated a score ≥ 1, (-) indicates a score of 0. On the left, the number of subjects with the same pattern of RBDSQ-q6 is reported.

	Dysautonomia by SCOPA-AUT			
# subjects	BL	YEAR 1	YEAR 2	YEAR 3
175	+	+	+	+
5	+	+	-	+
1	+	-	-	+
5	+	-	+	+
43	-	-	-	-
4	-	+	+	-
4	-	+	-	-
5	-	-	+	-
8	+	+	+	-
4	+	+	-	-
7	+	-	-	-
2	+	-	+	-
33	-	+	+	+
3	-	+	-	+
12	-	-	-	+
14	-	-	+	+

Supplementary Table 3.

Patients with PD were grouped based on their score of SCOPA-AUT across visits (BL to Year 3): (+) indicated a score ≥ 7, (-) indicates a score of <7. On the left, the number of subjects with the same pattern of SCOPA-AUT is reported.

Supplementary Table 4. Comparison across PD patients clustered based on pRBD and dysautonomia.

Pairwise comparison with Bonferroni correction was calculated to compare the scores of each trait between the four groups at BL (a) and year 3 (b). Clinical sub-groups were based on RBDSQ and SCOPA-AUT score at baseline, as detailed in the manuscript.

(a) BASELINE

Variable	group1	group2	p-value	adjusted p-value	adjusted p-value significance
UPDRS_total	Dys-pRBD-	Dys+pRBD+	1.69E-08	1.01E-07	****
	Dys-pRBD+	Dys-pRBD-	7.78E-01	1.00E+00	ns
	Dys-pRBD+	Dys+pRBD-	9.51E-04	6.00E-03	**
	Dys-pRBD+	Dys+pRBD+	2.22E-05	1.33E-04	***
	Dys-pRBD-	Dys+pRBD-	1.29E-05	7.74E-05	****
	Dys+pRBD-	Dys+pRBD+	1.15E-01	6.90E-01	ns
MOTOR_SYMPTOMS_updrs3	Dys-pRBD-	Dys+pRBD+	0.072	0.432	ns
	Dys-pRBD+	Dys-pRBD-	0.575	1	ns
	Dys-pRBD+	Dys+pRBD-	0.022	0.133	ns
	Dys-pRBD+	Dys+pRBD+	0.041	0.245	ns
	Dys-pRBD-	Dys+pRBD-	0.039	0.235	ns
	Dys+pRBD-	Dys+pRBD+	0.82	1	ns
MOTOR_SYMPTOMS_HY	Dys-pRBD-	Dys+pRBD+	0.015	0.091	ns
	Dys-pRBD+	Dys-pRBD-	0.476	1	ns
	Dys-pRBD+	Dys+pRBD-	0.456	1	ns
	Dys-pRBD+	Dys+pRBD+	0.296	1	ns
	Dys-pRBD-	Dys+pRBD-	0.047	0.28	ns
	Dys+pRBD-	Dysp+RBD+	0.695	1	ns
COGNITIVE_moca	Dys-pRBD-	Dys+pRBD+	0.234	1	ns
	Dys-pRBD+	Dys-pRBD-	0.083	0.497	ns
	Dys-pRBD+	Dys+pRBD-	0.175	1	ns
	Dys-pRBD+	Dys+pRBD+	0.01	0.057	ns
	Dys-pRBD-	Dys+pRBD-	0.549	1	ns
	Dys+pRBD-	Dys+pRBD+	0.056	0.339	ns
DYSAUTONOMIA_scopa_aut	Dys-pRBD-	Dys+pRBD+	1.01E-32	6.06E-32	****
	Dys-pRBD+	Dys-pRBD-	1.00E-03	6.00E-03	**
	Dys-pRBD+	Dys+pRBD-	6.98E-18	4.19E-17	****
	Dys-pRBD+	Dys+pRBD+	3.81E-18	2.29E-17	****
	Dys-pRBD-	Dys+pRBD-	1.09E-31	6.54E-31	****
	Dys+pRBD-	Dys+pRBD+	3.00E-03	1.80E-02	*
pRBD_rbdsq	Dys-pRBD-	Dys+pRBD+	2.70E-25	1.62E-24	****
	Dys-pRBD+	Dys-pRBD-	9.96E-11	5.98E-10	****
	Dys-pRBD+	Dys+pRBD-	1.65E-08	9.90E-08	****
	Dys-pRBD+	Dys+pRBD+	6.30E-02	3.79E-01	ns
	Dys-pRBD-	Dys+pRBD-	8.00E-03	4.90E-02	*
	Dys+pRBD-	Dys+pRBD+	9.08E-24	5.45E-23	****
ANXIETY_stai_trait	Dys-pRBD-	Dys+pRBD+	2.99E-06	1.79E-05	****
	Dys-pRBD+	Dys-pRBD-	1.80e-02	1.10e-01	ns
	Dys-pRBD+	Dys+pRBD-	7.92e-01	1.00E+00	ns

	Dys-pRBD+	Dys+pRBD+	1.64e-01	9.84e-01	ns
	Dys-pRBD-	Dys+pRBD-	4.00E-03	2.60E-02	*
	Dys+pRBD-	Dys+pRBD+	3.60E-02	2.18E-01	ns
DEPRESSION_gds	Dys-pRBD-	Dys+pRBD+	1.52E-07	9.12E-07	****
	Dys-pRBD+	Dys-pRBD-	6.19E-01	1.00E+00	ns
	Dys-pRBD+	Dys+pRBD-	1.71E-01	1.00E+00	ns
	Dys-pRBD+	Dys+pRBD+	1.00E-03	8.00E-03	**
	Dys-pRBD-	Dys+pRBD-	7.00E-03	4.10E-02	*
	Dys+pRBD-	Dys+pRBD+	5.00E-03	2.80E-02	*
PSYCHIATRIC_FEATURES_updrs1	Dys-pRBD-	Dys+pRBD+	2.13e-08	1.28e-07	****
	Dys-pRBD+	Dys-pRBD-	5.67e-01	1.00e+00	ns
	Dys-pRBD+	Dys+pRBD-	1.40e-02	8.40e-02	ns
	Dys-pRBD+	Dys+pRBD+	6.13e-04	4.00e-03	**
	Dys-pRBD-	Dys+pRBD-	2.00e-05	1.20e-04	***
	Dys+pRBD-	Dys+pRBD+	1.11e-01	6.66e-01	ns
DYSAUTONOMIA_SLEEP_updrs1	Dys-pRBD-	Dys+pRBD+	6.68e-14	4.01e-13	****
	Dys-pRBD+	Dys-pRBD-	9.20e-02	5.51e-01	ns
	Dys-pRBD+	Dys+pRBD-	7.41e-04	4.00e-03	**
	Dys-pRBD+	Dys+pRBD+	5.90e-06	3.54e-05	****
	Dys-pRBD-	Dys+pRBD-	3.78e-10	2.27e-09	****
	Dys+pRBD-	Dys+pRBD+	2.50e-02	1.48e-01	ns
DAILY_ACTIVITIES_updrs2	Dys-pRBD-	Dys+pRBD+	6.91e-14	4.15e-13	****
	Dys-pRBD+	Dys-pRBD-	1.10e-02	6.30e-02	ns
	Dys-pRBD+	Dys+pRBD-	7.60e-02	4.55e-01	ns
	Dys-pRBD+	Dys+pRBD+	4.74e-05	2.84e-04	***
	Dys-pRBD-	Dys+pRBD-	5.45e-07	3.27e-06	****
	Dys+pRBD-	Dys+pRBD+	3.00e-03	1.70e-02	*

(b) YEAR 3

Variable	group1	group2	p-value	adjusted p-value	adjusted p-value significance
UPDRS_total	Dys-pRBD-	Dys+pRBD+	6.17e-08	3.7e-07	****
	Dys-pRBD+	Dys-pRBD-	3.57e-01	1.0e+00	ns
	Dys-pRBD+	Dys+pRBD-	2.25e-01	1.0e+00	ns
	Dys-pRBD+	Dys+pRBD+	1.00e-03	6.0e-03	**
	Dys-pRBD-	Dys+pRBD-	4.00e-03	2.6e-02	*
	Dys+pRBD-	Dys+pRBD+	2.00e-03	1.3e-02	*
MOTOR_SYMPTOMS_updrs3	Dys-pRBD-	Dys+pRBD+	0.015	0.087	ns
	Dys-pRBD+	Dys-pRBD-	0.909	1.000	ns
	Dys-pRBD+	Dys+pRBD-	0.414	1.000	ns
	Dys-pRBD+	Dys+pRBD+	0.069	0.413	ns
	Dys-pRBD-	Dys+pRBD-	0.292	1.000	ns
	Dys+pRBD-	Dys+pRBD+	0.149	0.894	ns
MOTOR_SYMPTOMS_HY	Dys-pRBD-	Dys+pRBD+	0.005	0.028	*
	Dys-pRBD+	Dys-pRBD-	0.444	1.000	ns
	Dys-pRBD+	Dys+pRBD-	0.019	0.114	ns
	Dys-pRBD+	Dys+pRBD+	0.003	0.017	*
	Dys-pRBD-	Dys+pRBD-	0.036	0.215	ns
	Dys+pRBD-	Dysp+RBD+	0.569	1.000	ns
COGNITIVE_moca	Dys-pRBD-	Dys+pRBD+	0.000112	0.000672	***
	Dys-pRBD+	Dys-pRBD-	0.045000	0.268000	ns
	Dys-pRBD+	Dys+pRBD-	0.239000	1.000000	ns
	Dys-pRBD+	Dys+pRBD+	0.527000	1.000000	ns
	Dys-pRBD-	Dys+pRBD-	0.181000	1.000000	ns
	Dys+pRBD-	Dys+pRBD+	0.006000	0.037000	*
DYSAUTONOMIA_scopa_aut	Dys-pRBD-	Dys+pRBD+	4.22e-20	2.53e-19	****
	Dys-pRBD+	Dys-pRBD-	3.40e-02	2.04e-01	ns
	Dys-pRBD+	Dys+pRBD-	1.10e-02	6.40e-02	ns
	Dys-pRBD+	Dys+pRBD+	7.53e-09	4.52e-08	****
	Dys-pRBD-	Dys+pRBD-	6.58e-09	3.95e-08	****
	Dys+pRBD-	Dys+pRBD+	8.43e-08	5.06e-07	****
pRBD_rbdsq	Dys-pRBD-	Dys+pRBD+	1.78e-05	0.000107	***
	Dys-pRBD+	Dys-pRBD-	4.20e-02	0.250000	ns
	Dys-pRBD+	Dys+pRBD-	9.66e-01	1.000000	ns
	Dys-pRBD+	Dys+pRBD+	1.67e-01	1.000000	ns
	Dys-pRBD-	Dys+pRBD-	1.30e-02	0.080000	ns
	Dys+pRBD-	Dys+pRBD+	3.80e-02	0.227000	ns
ANXIETY_stai_trait	Dys-pRBD-	Dys+pRBD+	4.63e-11	2.78e-10	****
	Dys-pRBD+	Dys-pRBD-	4.30e-02	2.60e-01	ns
	Dys-pRBD+	Dys+pRBD-	3.46e-01	1.00e+00	ns
	Dys-pRBD+	Dys+pRBD+	4.00e-03	2.30e-02	*
	Dys-pRBD-	Dys+pRBD-	5.79e-05	3.47e-04	***
	Dys+pRBD-	Dys+pRBD+	2.00e-03	1.40e-02	*
DEPRESSION_gds	Dys-pRBD-	Dys+pRBD+	2.05e-09	1.23e-08	****
	Dys-pRBD+	Dys-pRBD-	6.40e-02	3.82e-01	ns
	Dys-pRBD+	Dys+pRBD-	3.83e-01	1.00e+00	ns
	Dys-pRBD+	Dys+pRBD+	6.00e-03	3.30e-02	*
	Dys-pRBD-	Dys+pRBD-	3.65e-04	2.00e-03	**
	Dys+pRBD-	Dys+pRBD+	1.20e-02	7.00e-02	ns
PSYCHIATRIC_FEATURES_updrs1	Dys-pRBD-	Dys+pRBD+	6.74e-09	4.04e-08	****
	Dys-pRBD+	Dys-pRBD-	9.61e-01	1.00e+00	ns
	Dys-pRBD+	Dys+pRBD-	5.00e-03	3.00e-02	*
	Dys-pRBD+	Dys+pRBD+	1.69e-05	1.01e-04	***
	Dys-pRBD-	Dys+pRBD-	9.88e-05	5.93e-04	***
	Dys+pRBD-	Dys+pRBD+	4.00e-03	2.60e-02	*

**DYSAUTONOMIA_SLEEP_updrs1**	Dys-pRBD-	Dys+pRBD+	3.01e-11	1.81e-10	****
	Dys-pRBD+	Dys-pRBD-	1.20e-02	7.20e-02	ns
	Dys-pRBD+	Dys+pRBD-	7.88e-01	1.00e+00	ns
	Dys-pRBD+	Dys+pRBD+	6.59e-04	4.00e-03	**
	Dys-pRBD-	Dys+pRBD-	7.65e-04	5.00e-03	**
	Dys+pRBD-	Dys+pRBD+	9.90e-05	5.94e-04	***

	GI (*p*)	UROLOGIC (*p*)	CV (*p*)	THERM (*p*)	PM (*p*)	SEX (*p*)	GI* pRBD (*p*)	URO* pRBD (*p*)	CV* pRBD (*p*)	THERM* pRBD (*p*)	PM* pRBD (*p*)	SEX* pRBD (*p*)
**MDS-UPDRS Parts 1 (dys_RBD)**	**<0.001*****	**<0.001*****	**<0.001*****	0.239	0.495	0.742	0.805	0.786	0.702	0.508	0.240	0.777
**MDS-UPDRS Parts 1 (psychiatric)**	0.217	0.482	0.419	0.314	0.136	**0.006****	0.298	0.483	0.832	0.155	0.128	0.127
**MDS-UPDRS Parts 2**	**<0.001*****	0.120	0.101	0.687	**0.024***	0.949	0.411	0.198	0.906	0.227	0.134	0.435
**MDS-UPDRS Part 3**	**0.015***	0.739	0.567	0.166	**0.003****	0.363	0.732	0.236	0.990	**0.039***	**0.001*****	0.379
**MDS-UPDRS Total**	**<0.001*****	0.116	0.065	0.548	**0.001*****	0.818	0.997	0.174	0.879	**0.023***	**0.001*****	0.614
**H&Y**	0.141	0.075	0.100	0.584	**0.017***	**0.005****	0.898	0.379	0.169	0.227	**0.026***	0.835
**STAI (anxiety)**	0.070	0.936	**0.031***	0.110	0.987	0.757	0.470	0.109	0.636	0.849	0.943	0.281
**GDS (depression)**	0.236	0.686	0.352	0.446	0.062	0.492	0.748	0.088	0.256	0.411	0.120	0.144
**MoCA (cognitive)**	0.304	0.494	0.350	0.506	0.123	**0.017***	0.838	0.410	0.987	0.611	0.450	0.055

Supplementary Table 5. Correlation of SCOPA-AUT scale sub-scores and progression of motor and non-motor PD-related symptoms.

Mixed-model for multiple regression was used to assess the contribution of sub-scores for autonomic features and of the interaction between each trait and pRBD score on motor (MDS-UPDRS part 3, H&Y, MDS-UPDRS part 2) and non-motor symptoms (cognitive – MoCA -, dysautonomia - MDS-UPDRS part 1 (dys_RBD), psychiatric symptoms – depression (GDS scale), anxiety (STAI-TRAIT), and MDS-UPDRS part 1 (psychiatric)), and MDS-UPDRS total score, across visits (BL-Y3). pRBD: binary score (subjects with RBDSQ-q6 = 0 at all visits vs subjects with at least one visit with score >1). GI: gastrointestinal; CV: cardiovascular; THERM: thermoregulatory; PM: pupillomotor; SEX: sexual. ***< 0.001; **<0.01; *<0.05.
